# Drug-Resistant Tuberculosis in Rural Eastern Cape, South Africa: A Study of Patients’ Characteristics in Selected Healthcare Facilities

**DOI:** 10.3390/ijerph21121594

**Published:** 2024-11-30

**Authors:** Lindiwe Modest Faye, Mojisola Clara Hosu, Teke Apalata

**Affiliations:** Department of Laboratory Medicine and Pathology, Walter Sisulu University, Private Bag X5117, Mthatha 5099, South Africa; mhosu@wsu.ac.za (M.C.H.); tapalata@wsu.ac.za (T.A.)

**Keywords:** drug-resistant tuberculosis, comorbidities, socioeconomic factors, rural healthcare, treatment outcomes, HIV co-infection and mortality rates

## Abstract

This study investigated the characteristics and outcomes of drug-resistant tuberculosis patients in selected rural healthcare facilities in the Eastern Cape, South Africa. A retrospective review of clinical records from 456 patients, covering the period from January 2018 to December 2020, revealed a statistically significant relationship between DR-TB types and age groups (Chi-square statistic: 30.74, *p*-value: 0.015). Younger adults (19–35 years) and middle-aged adults (36–50 years) are more frequently affected by RR-TB and MDR-TB, which are the most prevalent forms of DR-TB. Less common types, including Pre-XDR, XDR, and INH TB, were observed in smaller numbers. The study suggests that DR-TB imposes a heavy burden on the working age population. Gender analysis shows that while the frequency of DR-TB differs between males and females, the percentage distribution of DR-TB types is relatively equal. Both genders are predominantly affected by RR-TB and MDR-TB, which together account for nearly 90% of cases. Pre-XDR, XDR, and INH-resistant TB are much less common, comprising only a small percentage of cases in both males and females. High-risk behaviors such as smoking and drinking are linked to a wider diversity of DR-TB types, while occupations like mining and prison work show higher rates of RR-TB and MDR-TB. In HIV-positive individuals, DR-TB is more common, but the distribution of DR-TB types between HIV-positive and negative groups shows no statistically significant difference. However, HIV-positive individuals have a 20% lower survival rate (65%) compared to HIV-negative patients (85%). Financial stability and comorbidities also significantly influence outcomes, with patients having stable income and fewer high-risk comorbidities experiencing better survival and treatment outcomes. The findings underscore the importance of addressing socioeconomic disparities and strengthening healthcare infrastructure to improve DR-TB treatment outcomes in rural Eastern Cape.

## 1. Introduction

The global strategy to end the TB epidemic by 2030 is crucial as TB remains a leading infectious cause of death worldwide, causing approximately 1.4 million deaths annually [1]. Despite the progress in reducing the disease burden, TB, HIV, and STIs continue to be major public health challenges in South Africa, particularly among key and vulnerable populations. In response, South Africa launched the 5th National Strategic Plan (NSP) 2023–2028, “The People’s NSP”, to integrate treatment for TB, HIV, and STIs with the goal of eradicating TB as a public health threat by 2030 [2,3].

TB is a chronic infectious disease caused by *Mycobacterium tuberculosis*, primarily affecting the lungs but capable of impacting other body parts [4,5,6,7]. It spreads through airborne droplets when an infected person coughs or sneezes [8]. South Africa remains among the countries with the highest TB burden, including cases of drug-resistant TB (DR-TB) and TB-HIV coinfections, reporting over 322,000 new TB cases annually, with 4.4% being drug-resistant [9]. While drug-susceptible TB cases have decreased, MDR-TB cases are on the rise, complicating TB control efforts [2,10,11].

DR-TB strains include rifampicin mono-resistant TB (RMR-TB), rifampicin-resistant TB (RR-TB), isoniazid-resistant TB (IR-TB), MDR-TB, pre-extensively drug-resistant TB (pre-XDR-TB), and extensively drug-resistant TB (XDR-TB) [12,13]. RMR-TB is resistant to rifampicin but remains susceptible to isoniazid, while RR-TB is resistant to rifampicin irrespective of resistance to other drugs. MDR-TB, resistant to both rifampicin and isoniazid, is more difficult and costly to treat [13,14,15,16]. Pre-XDR-TB is resistant to rifampicin and any fluoroquinolone, and XDR-TB, first identified in KwaZulu-Natal in 2005, exhibits resistance to rifampicin, fluoroquinolones, and key second-line drugs [10,17]. Cases of totally drug-resistant (TDR) TB strains were reported in Eastern Cape Province between 2008 and 2009 [10].

Patient characteristics such as gender, age, socioeconomic status, and level of education have been observed to determine healthcare-seeking behavior among TB patients [18,19] and contribute to delayed treatment initiation [20]. TB patients in rural areas often have lower educational levels, which impacts their understanding of TB symptoms, treatment adherence, and health-seeking behavior [21]. They experience poorer treatment outcomes, including higher mortality rates and greater loss to follow-up compared to patients in urban settings [21,22]. Financial constraints, transportation issues, and food insecurity often hinder patients’ adherence to treatment, despite the availability of free TB medications [23,24].

Vulnerable populations, including miners, prison inmates, and those in overcrowded conditions, are at a heightened risk of TB [25,26]. Inmates face overcrowding, poor ventilation, and inadequate sanitation, contributing to high TB transmission rates [27,28]. HIV-positive individuals are also at an elevated risk, being up to 20 times more likely to develop TB compared to HIV-negative individuals [29].

Eastern Cape, among South Africa’s poorest provinces, reflects the profound impact of poverty on TB prevalence [30,31]. Poverty exacerbates malnutrition, overcrowding, and limited access to healthcare, all of which facilitate TB transmission and complicate treatment [32,33]. These challenges are worsened by underfunded and overburdened healthcare systems that lack sufficient staffing, medical supplies, and effective TB management strategies [34]. The resulting inadequate care further contributes to high noncompliance rates among TB patients, including those with drug-resistant forms [35,36].

Understanding patient demographics, socioeconomic status, and challenges faced in accessing and adhering to TB treatment can inform better strategies for TB control in resource-limited settings. This study aimed to profile the characteristics of DR-TB patients in selected rural health facilities in Eastern Cape, South Africa.

## 2. Materials and Methods

### 2.1. Study Design

The study involved a retrospective review of clinical records of DR-TB patients enrolled for treatment in six healthcare facilities between January 2018 and December 2020. Clinical records of DR-TB patients including socio-demographics, clinical, laboratory, and treatment-specific data, were documented. The HIV testing results and co-administration for comorbidity, and the presence of co-morbid conditions, and treatment outcomes were recorded.

### 2.2. Study Setting

The study population included patients diagnosed with DR-TB in six participating facilities, who met the inclusion criteria of a bacteriologically confirmed diagnosis of DR-TB, MDR-TB or XDR-TB. Bacteriological confirmation diagnosis was performed at National Health Laboratory Services—TB laboratory in Nelson Mandela Academic Hospital, Mthatha, following manufacturer’s instructions for three procedures Xpert^®^ MTB/RIF assay, phenotypic drug susceptibility testing—automated BACTEC Mycobacterial Growth Indicator Tube 960 (Becton Dickinson, New York, NY, USA) and Genotypic DST—GenoType MTBDR plus version 2.0 (Hain Life science, Nehren, Germany). The researchers purposely and conveniently selected 5 DR-TB clinics and a referral hospital that offers DR-TB, MDR-TB, and XDR-TB management services to these clinics.

### 2.3. Data Collection Methods

We conducted a retrospective review of patients’ + clinic files with DRTB from healthcare facilities. Eligible patients had confirmed DRTB, HIV co-infection, comorbidities and a treatment outcome registered between 2018 and 2020. Socio-demographic and clinical characteristics and TB treatment outcomes were included in the data collection.

### 2.4. Data Analysis Methods

Proportion (%) was calculated when data were categorical while mean or median (±standard deviation) was computed when data was continuous. Student’s *t*-test was performed to assess the differences between two means and ANOVA between groups. Either the Chi-square test with and without trend or Fischer’s exact test was used to test the degree of association of categorical variables. The Chi-square test (for categorical comparisons) or ANOVA (for comparing group means) were applied to test for significance. ANOVA assumes that the data follow a normal distribution and have homogeneity of variance. If the initial tests (Chi-Square or ANOVA) show significant results, post-hoc pairwise comparison tests were used to identify which specific groups are significantly different from each other. In this study, ChatGPT version 2, developed by OpenAI, was used for data analysis and visualization, in addition to this, python version 3.8. and R version 4.1.1 software were also used. A *p* < 0.05 was considered to be significant.

### 2.5. Definition of Terms

MDR-TB is defined as TB caused by the *M. tuberculosis* strain that is resistant to at least isoniazid and rifampin.

Pre-XDR-TB is defined as TB caused by the *M. tuberculosis* strains that fulfil the definition of MDR/RR-TB and are also resistant to any fluoroquinolone.

XDR-TB is defined as TB caused by the *M. tuberculosis* strains that fulfil the definition of MDR/RR-TB and are also resistant to any fluoroquinolones and at least one additional Group A drug (levofloxacin or moxifloxacin, bedaquiline and linezolid).

DG refers to individuals receiving disability grants, often associated with underlying health conditions.

UIF refers to patients receiving benefits from the unemployment insurance fund.

## 3. Results

There were 456 patients with a mean age of 37.5 years (SD = 14.9 years). The ages ranged from 1 year to 86 years, with the 25th percentile at 27.75 years, the median age at 36 years, and the 75th percentile at 47 years. This indicates that while the majority of patients are between 27.75 and 47 years old, the age distribution is moderately spread out, with a slight skew towards older ages.

**Figure 1:**
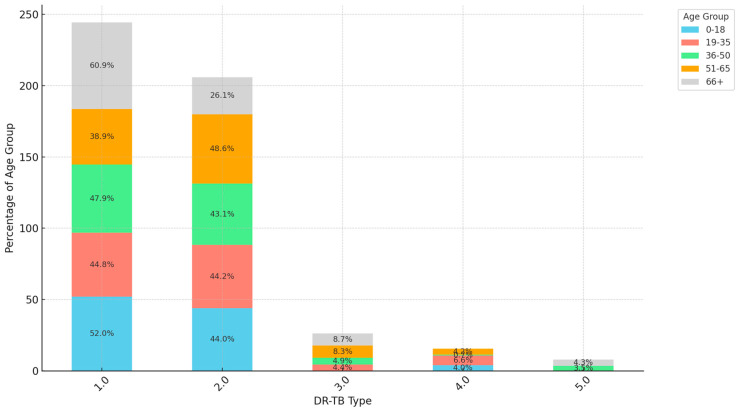
Distribution of DR-TB type by age group. (1.0 = RR, 2.0 = MDR, 3.0 = Pre-XDR, 4.0 = XDR, 5.0 = INH-R).

Figure 1 shows that there is a statistically significant difference in the distribution of DR-TB types across different age groups (Chi-square statistic (χ^2^): 30.74, *p*-value: 0.015). The types of DR-TB are associated with age groups in a statistically significant way, indicating that certain age groups may be more or less likely to have specific types of DR-TB. RR-TB has the highest representation in the age group 0–18, accounting for 52% of cases, followed by the age groups 19–35 and 36–50. The older age groups, 51–65 and 66+, show smaller proportions for this DR-TB type. In contrast, MDR-TB is led by the age group 19–35, which accounts for 44.2%, closely followed by the 0–18 age group at 44.0%. The remaining age groups, particularly 36–50, contribute smaller but still noticeable proportions to this MDR-TB. For pre-XDR-TB, XDR-TB and INH-TB, the overall incidence is significantly lower compared to RR-TB and MDR-TB. The 66+ age group has a slightly higher proportion for pre-XDR-TB at 8.7%, although this may be reflective of a smaller sample size, making the DR-TB type less prevalent across all age groups. The age distribution shows a heavy burden of DR-TB on working-age adults, particularly those aged 19–35, indicating a significant impact on this population demographic.

**Figure 2:**
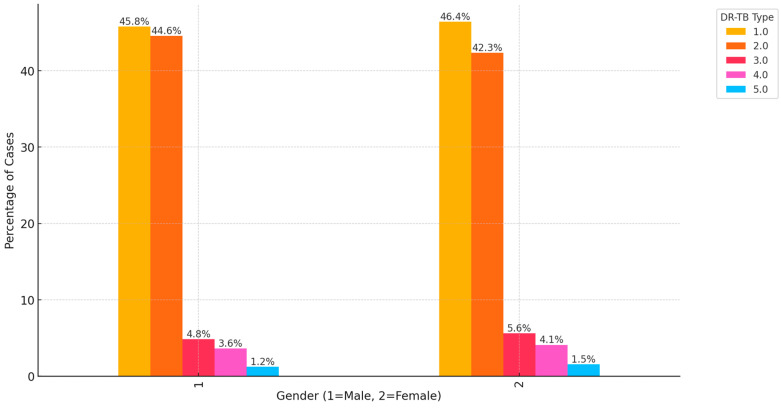
Distribution of DR-TB type by gender. (1.0 = RR, 2.0 = MDR, 3.0 = Pre-XDR, 4.0 = XDR, 5.0 = INH-R).

Figure 2 below displays the distribution of different DR-TB types across genders. RR-TB and MDR-TB dominate the overall distribution for both genders. Among males, 45.8% of DR-TB cases were RR-TB, and 44.6% were MDR-TB. Together, these two types account for over 90% of DR-TB cases among males. Similarly, among females, 46.4% of DR-TB cases were RR-TB, and 42.3% were MDR-TB, making up the vast majority (over 88%) of DR-TB cases in females. This suggests that both males and females are predominantly affected by RR-TB and MDR-TB, with minimal variation between the genders. In contrast, Pre-XDR, XDR-TB, and INH-R TB are much less common in both males and females. Specifically, Pre-XDR represents 4.8% of male cases and 5.6% of female cases, XDR-TB accounts for 3.6% of male cases and 4.1% of female cases and INH-R TB is seen in 1.2% of male cases and 1.5% of female cases. This indicates that the more advanced DR-TB types (Pre-XDR, XDR-TB and INH-R TB) are much less prevalent in the overall DR-TB case pool, though females show slightly higher proportions in Pre-XDR and XDR-TB.

**Figure 3:**
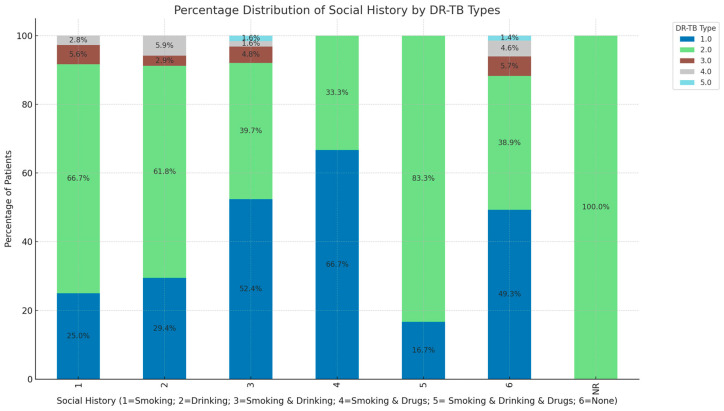
Distribution of different DR-TB types based on patients’ social history. (1 = Smoking; 2 = Drinking; 3 = Smoking & Drinking; 4 = Smoking & Drugs; 5 = Smoking, Drinking & Drugs; 6 = None, NR = not reported; 1.0 = RR, 2.0 = MDR, 3.0 = Pre-XDR, 4.0 = XDR, 5.0 = INH-R).

Figure 3 shows the analysis of DR-TB types by social history which reveals a significant trend that can inform targeted public health interventions. Among patients with no recorded social behaviors (smoking, drinking, or drug use), indicated in column 6 of the bar chart, 49.3% have RR-TB and 38.9% have MDR-TB, indicating that these TB types are prevalent even in the absence of known social risk factors. Social behaviors such as smoking and drinking alone are strongly associated with MDR-TB, comprising 66.7% of the smoking group and 61.8% of the drinking group, with RR-TB accounting for 25.0% and 29.4%, respectively. In contrast, more complex behavior combinations, such as “Smoking & Drinking” and “Smoking & Drugs”, demonstrate balanced associations, with 52.4% to 66.7% of cases involving RR-TB and 39.7% to 33.3% involving MDR-TB. The “Smoking & Drinking & Drugs” category shows the highest percentage of MDR TB at 83.3%, suggesting that multiple risk behaviors are more closely linked to RR TB. Less common DR-TB types, such as Pre-XDR and XDR, appear at lower percentages across all social history categories, with isolated occurrences like 5.9% XDR in the “Drinking” group and 4.6% in the “None” category. This indicates these types are less influenced by the analyzed social factors but are still present.

**Figure 4:**
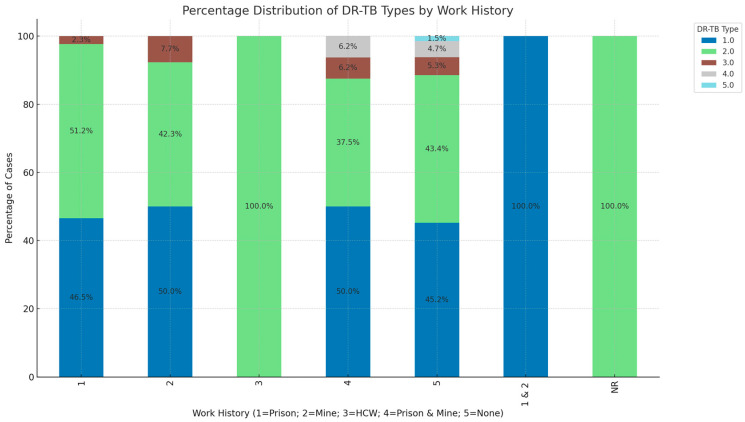
Comparison of DR-TB type and patient work history. DR-TB type: 1.0 = RR, 2.0 = MDR, 3.0 = Pre-XDR, 4.0 = XDR, 5.0 = INH-R.

The DR-TB types by work history reveal distinct patterns. In the “Prison” category (1), 46.5% of cases are RR-TB and 51.2% are MDR-TB, with minor representations of pre-XDR and XDR-TB. The “Mine” category (2) shows 50.0% of cases as RR-TB and 42.3% as MDR-TB, along with 7.7% being Pre-XDR. The “HCW” category (3) stands out with 100.0% of cases as MDR-TB. In the “Prison & Mine” combined category (4), 50.0% of cases are RR-TB, 37.5% are MDR-TB, and Pre-XDR and XDR-TB both account for 6.2%. Finally, in the “None” category (patients who never worked/unemployed) (5), 45.2% are RR-TB, 43.4% are MDR-TB, with smaller proportions for other DR-TB types. The “NR” category represents people with no recorded work history (Figure 4).

**Figure 5:**
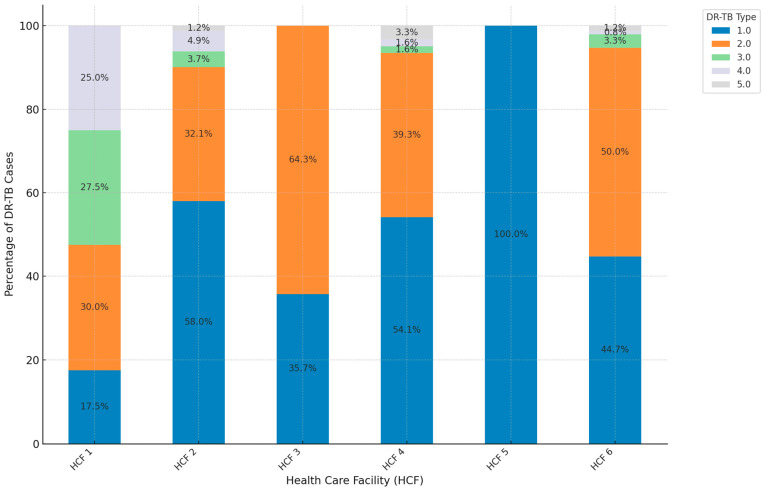
Proportions of DR-TB types by clinic. (1.0 = RR, 2.0 = MDR, 3.0 = Pre-XDR, 4.0 = XDR, 5.0 = INH-R).

Figure 5 compares the proportions of different types of DR-TB across six healthcare facilities (HCFs). RR-TB was more common in HCF 2, 4 and 5 with proportions of 58.0%, 54.1% and 100% respectively. The concentration of MDR-TB was seen more in HCF 1, 3, and 6 with proportions of 30.0%, 64.3%. and 50.0% respectively. Pre-XDR was found in 4 HCFs in smaller proportions but HCF 1 had 27.5% occurrence. A few facilities show small but significant proportions of the rarer XDR, and INH-R TB, with 0.8–25.0% of cases in these types. This distribution might indicate unique resistance patterns within these facilities or specific community characteristics that increase exposure to these less common DR-TB types. Lastly, facilities with a broader range of DR-TB types likely serve a diverse community, suggesting multiple exposure sources.

**Figure 6:**
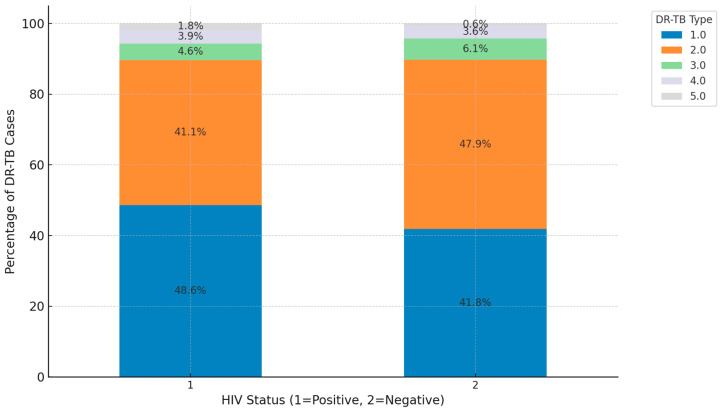
Distribution of DR-TB types within each HIV status. (1.0 = RR, 2.0 = MDR, 3.0 = Pre-XDR, 4.0 = XDR, 5.0 = INH-R).

The distribution of DR-TB types by HIV status reveals key differences in prevalence. Among HIV-positive patients (1), 48.6% have RR-TB, 41.1% MDR-TB, 4.6% have Pre-XDR, 3.9% have XDR-TB, and 1.8% are cases of INH-R-TB. In contrast, for HIV-negative patients (2), 41.8% have RR-TB, 47.9% have MDR-TB, 6.1% are Pre-XDR, 3.6% are XDR-TB, and 0.6% are cases of INH-R-TB. This indicates that MDR-TB is more prevalent among HIV-negative patients, while RR-TB is more common among HIV-positive patients. Although Pre-XDR and XDR types occur in both groups, XDR-TB appears at a slightly higher percentage in HIV-negative individuals (Figure 6).

**Figure 7:**
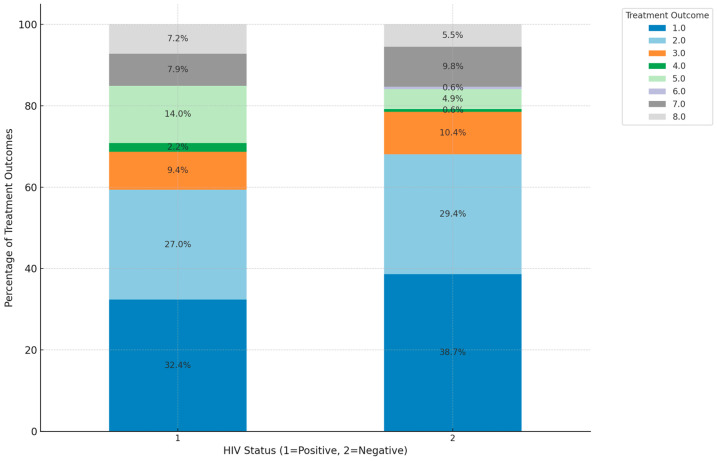
Survival rates for TB and HIV co-infection. (Treatment outcomes: 1 = cured: 2 = treatment completed: 3 = LTFU: 4 = treatment failed: 5 = died; 6 = moved out; 7 = transferred out; 8 = still on treatment).

For HIV-positive patients (status 1), cured and treatment-completed outcomes together account for approximately 60% of cases, indicating that more than half of co-infected individuals reached favorable outcomes. Mortality (died) is notably high, with about 14.0% of HIV-positive patients succumbing to the infection, reflecting a significant mortality risk within this group. Loss to follow-up (LTFU) and treatment failure together represent around 11.6% of cases, highlighting a subset of patients who either did not complete treatment or did not respond effectively. In HIV-negative patients (Status 2), cured and treatment-completed outcomes are higher, comprising 68.1% of cases, showing a more favorable prognosis compared to HIV-positive individuals. Mortality is lower in this group, at around 5.0%, underscoring the increased death risk associated with HIV co-infection. LTFU and treatment failure represent about 11.0% of cases, indicating fewer challenges in maintaining effective treatment among HIV-negative patients. This analysis illustrates the impact of HIV on TB survival rates, with co-infected patients facing higher mortality and slightly lower treatment success rates compared to their HIV-negative counterparts. (Figure 7).

**Figure 8:**
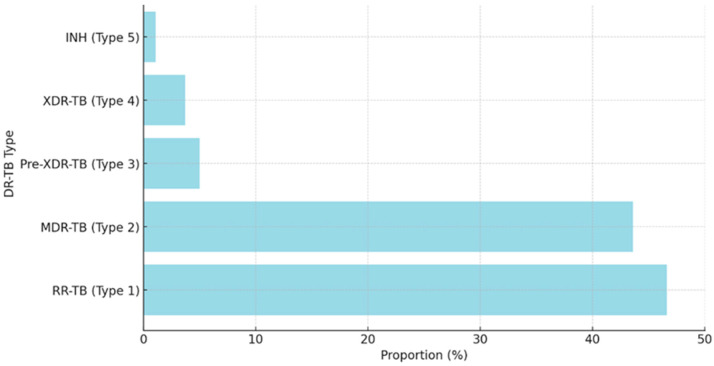
The proportions of DR-TB types in patients with comorbidities.

In Figure 8 below, the majority of DR-TB cases are either RR-TB or MDR-TB, together accounting for approximately 85% of the total cases. Pre-XDR-TB, XDR-TB, and INH-resistant TB are less common, with each making up a smaller proportion of the cases.

**Figure 9:**
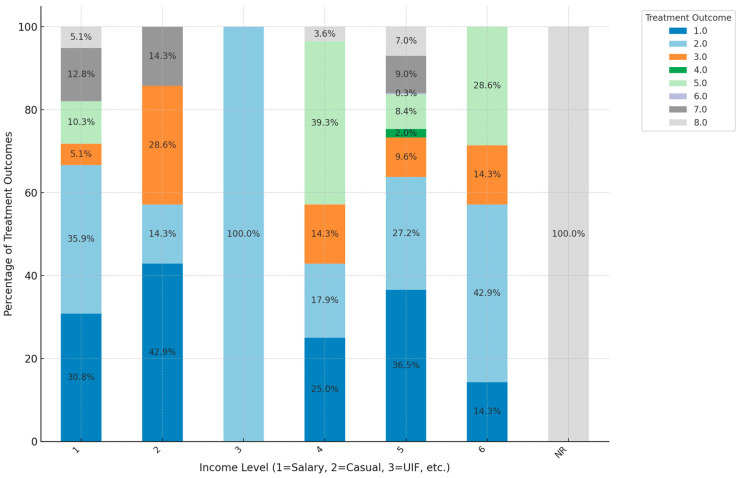
Impact of income on treatment outcomes. (1 = Salary or Wages; 2 = Casual; 3 = UIF; 4 = DG; 5 = No income; 6 = Self-employed, NR = not reported; Treatment outcomes: 1 = Cured: 2 = Treatment completed: 3 = LTFU: 4 = Treatment failed: 5 = Died; 6 = Moved out; 7 = transferred out; 8 = still on treatment).

In salary or wages (Category 1), cured and treatment-completed outcomes dominate, making up 73.3% of cases, suggesting that patients with regular income from salaries or wages have a higher likelihood of successful treatment. Mortality (Died) and LTFU are low in this group, both totaling 15.4%, indicating that income stability may contribute to treatment adherence and reduced mortality. In casual income (Category 2), successful outcomes are slightly lower than in salaried patients, with cured and treatment-completed accounting for 57.2%. For those receiving unemployment insurance fund (UIF, Category 3), success rates are moderate, with no patient cured, but they all completed treatment. In the disability grants (DG, Category 4) group, successful outcomes are lower, with cured and treatment completed at 42.9%. Mortality is relatively high in this group, at 39.3%, highlighting challenges in managing treatment among those on disability support. In the no income (Category 5) group, a moderate success rate is observed, with cured and treatment-completed outcomes at 63.7%. Mortality is 8.4%, and LTFU cases also make up about 10.0%, indicating that lack of income is strongly associated with poorer outcomes. For the self-employed (Category 6) group, successful outcomes are similar to casual income patients, with cured and treatment-completed outcomes at about 57.2%. Mortality and LTFU rates are moderate, each representing 42.9%, suggesting some variability in outcomes due to self-employment. This analysis underscores that stable income (salary or wages) is linked to higher treatment success and lower mortality, while lower or inconsistent income levels are associated with higher mortality and LTFU rates, emphasizing the role of financial stability in effective TB treatment outcomes (Figure 9).

**Figure 10:**
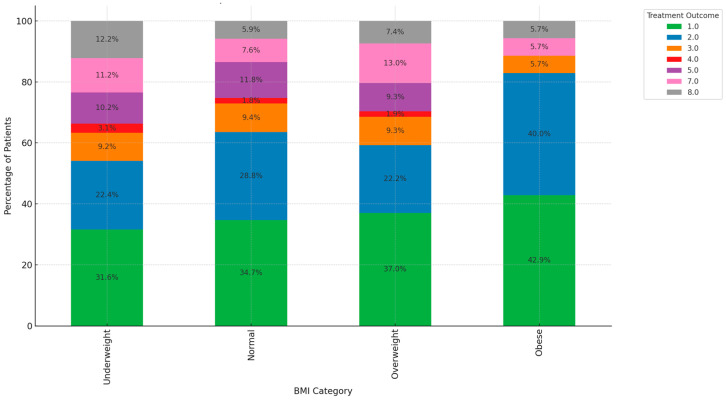
Comparison of BMI and treatment outcomes. (Treatment outcomes: 1 = cured: 2 = treatment completed: 3 = LTFU: 4 = treatment failed: 5 = died; 6 = moved out; 7 = transferred out; 8 = still on treatment).

Figure 10 shows a comparison of BMI and treatment outcomes which reveals notable trends across different BMI categories. Underweight patients face significant challenges, with only 31.6% categorized as cured and 22.4% completing treatment. They exhibit higher unfavorable outcomes, with 9.2% LTFU and 3.1% still on treatment. Normal BMI patients show a more balanced outcome distribution, with 34.7% cured and 28.8% completing treatment. However, 9.4% died, and 7.6% were transferred out, indicating that some risks remain in this group. Overweight patients demonstrate a higher success rate, with 37.0% cured and 22.2% completing treatment. Despite better outcomes, 9.3% died and 13.0% were transferred out. Obese patients have the highest proportion of positive outcomes, with 42.9% cured and 40.0% completing treatment, coupled with fewer adverse results, indicating a stronger overall treatment success.

The analysis reveals significant disparities in death rates across different age groups and income categories, highlighting a pattern of vulnerability, particularly among disability grant (DG) recipients. DG recipients consistently show elevated death rates across multiple age groups: 50% in the 19–35 age group, 33.3% in the 36–50 group, and 44.4% in the 51–65 group. This suggests that individuals receiving a disability grant may face increased mortality risks, likely due to underlying health conditions associated with disability.

**Figure 11:**
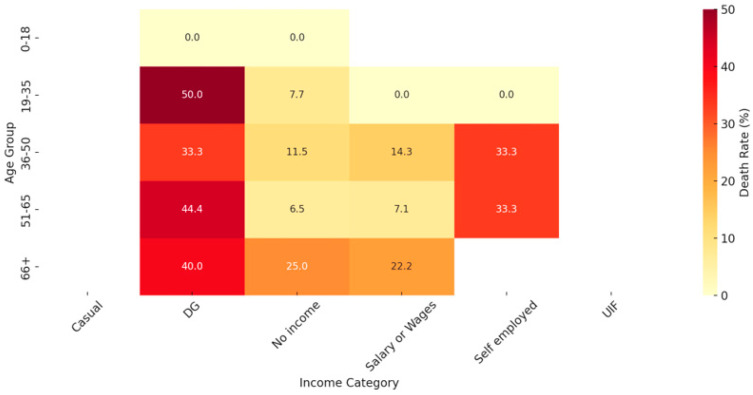
Death rate by age group and income category.

In the 19–35 age group, the high death rate of 50% among DG recipients is particularly striking, indicating a heightened risk for younger individuals with disabilities. In contrast, other income categories within this group, such as salary or wages and self-employed, show relatively low or zero death rates, suggesting that income stability and fewer health issues may offer some protection. The 36–50 age group shows high death rates in both self-employed (33.3%) and DG categories (33.3%), likely due to the onset of more chronic health conditions compared to younger groups. The no income category also shows a notable death rate of 11.5%, though lower than DG and self-employed, indicating potential economic vulnerability within this age range. In the 51–65 age group, DG recipients (44.4%) and self-employed individuals (33.3%) again experience high death rates, underscoring the combined impact of aging and economic stress on mortality risk. Even the no income category shows a death rate of 6.5%, highlighting moderate vulnerability in this age bracket. Among the senior age group (66+), death rates are particularly high for individuals with no income (25%) and those in the DG category (40%). This pattern underscores the substantial mortality risk associated with economic instability in old age, where lack of income correlates with increased vulnerability. Interestingly, the salary or wages category shows a comparatively lower death rate (22.2%) than DG, suggesting that income stability in old age may have a protective effect. In the younger age groups (19–35 and 36–50), individuals with stable income (salary or wages) and self-employment show lower death rates compared to those with no income or receiving DG (social grant), suggesting that financial stability may protect against the worst outcomes. There is an increased vulnerability as the age increases, the protective effect of income appears to diminish, with deaths occurring across all income categories, especially among the elderly (51+), indicating that age-related factors may outweigh the benefits of financial stability (Figure 11).

**Figure 12:**
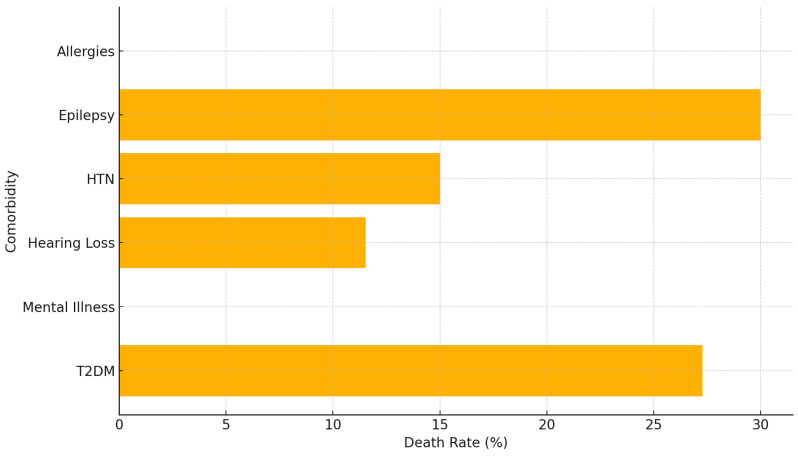
Death rate by specific comorbidity.

High-risk comorbidities like epilepsy and T2DM have the highest death rates, nearly 30%, making DR-TB patients with these conditions particularly vulnerable while moderate-risk comorbidities like mental illness and hypertension present moderate risks, with death rates between 15% and 25%, requiring significant attention in DR-TB management. The lower-risk comorbidities like allergies and hearing loss have relatively lower death rates (~10%), indicating that these conditions, while risky, are less critical compared to epilepsy, T2DM, and mental illness (Figure 12).

**Figure 13:**
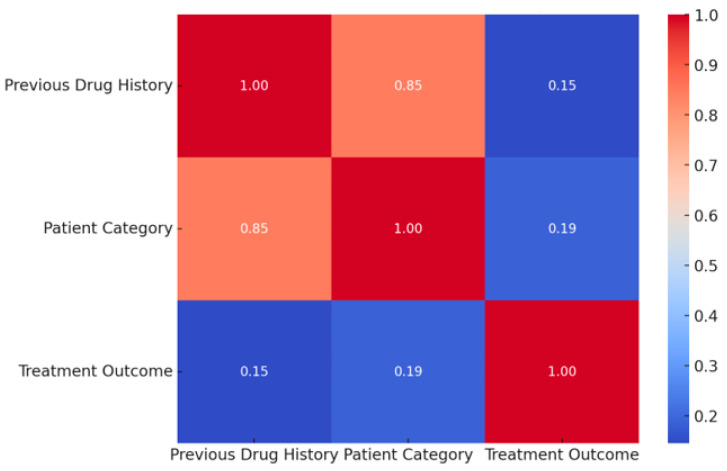
Relationship between previous drug history, patient category, and treatment outcome.

There is a strong positive correlation (0.85) between previous drug history and patient category, suggesting that prior treatment experience is a strong factor in defining patient category. A weak positive correlation (0.15) between previous drug history and treatment outcome indicates a slight but not strong association, patients with a history of drug treatment may have somewhat lower success rates or are more likely to experience treatment challenges, though this is not a definitive predictor of outcomes. The weak positive correlation (0.19) suggests a minor association between a patient’s category and their treatment outcome (Figure 13).

## 4. Discussion

Tuberculosis patients in rural areas face a complex interplay of socioeconomic disadvantages, healthcare access issues, and demographic trends that significantly impact their treatment outcomes and overall health. Age has a significant impact on TB treatment outcomes, influencing factors such as treatment efficacy, adherence, recovery rates, and the likelihood of complications. Studies indicate that the risk of unsuccessful TB treatment outcomes increases with age. Specifically, older patients (aged above 45 years) tend to have lower treatment success rates compared to younger patients. This is attributed to higher default rates, increased mortality, and the presence of comorbidities that complicate treatment [37]. A study by [38] found that patients aged under 20 years had 84% lower likelihood of achieving successful treatment outcomes compared to those aged above 60. This finding suggests that younger patients engage in riskier behaviors (e.g., substance abuse) that negatively impact their treatment adherence and outcomes. Children generally have less developed immune systems, which can make them more vulnerable to severe forms of TB. However, younger patients often respond well to treatment if it is started early and managed effectively.

RR-TB and MDR-TB were the most prevalent TB types in our study, each with over 200 cases, mainly affecting patients aged 19–35 and 36–50. These types were less common in the 0–18 and 66+ age groups. Individuals aged 19–50 had higher exposure to TB due to their active social and economic involvement in environments like workplaces and public transportation, increasing their risk of contracting drug-resistant strains [39]. In contrast, the 0–18 and 66+ age groups have lower exposure, as younger individuals are often under guardians’ care and older adults are more likely to be retired or less mobile, reducing their risk [40]. Pre-XDR-TB, XDR-TB, and INH-resistant TB are less common overall but predominantly affect the 19–35 and 36–50 age groups due to their higher exposure to TB, challenges with treatment adherence, and delayed healthcare access. Research indicates that pre-XDR-TB cases are more frequently observed in young adults aged 15–34, with one study specifically noting that 70% of these cases occur within this age group [41,42]. The higher prevalence of DR-TB in the 19–35 and 36–50 age groups is likely due to several factors like stress, malnutrition, and co-existing conditions like HIV, which can weaken their immune systems, making it harder to combat TB infections [13,40]. Inconsistent treatment adherence, driven by work commitments and limited healthcare access, further contributes to the development of DR-TB strains [41]. Economic pressures, migration, urbanization, and occupational risks in this demographic also play significant roles in the spread and worsening of DR-TB [42].

The social history of patients with DR-TB in this study highlights the significant impact of socioeconomic factors on treatment outcomes. Patients from lower socioeconomic backgrounds, especially those receiving social grants/DG or without stable income, face poorer outcomes, including higher rates of LTFU and mortality. Economic instability contributes to inadequate access to healthcare, poor living conditions, and insufficient nutrition, all of which exacerbate the challenges of managing DR-TB [43,44]. Income stability plays a crucial role in treatment outcomes for TB patients. Those with stable or higher income sources, such as salaries or self-employment, are more likely to achieve successful outcomes, including being cured or completing treatment which is corroborated by the studies of Tadokera et al. and Cramm et al. [45,46]. Individuals with stable incomes are known to afford healthcare services, such as regular doctor visits, diagnostic tests, and medications, allowing them to adhere to treatment plans without interruptions caused by financial constraints [46]. Additionally, higher income enables timely healthcare seeking, which is crucial for early diagnosis and effective TB treatment, reducing the risk of complications and improving the chances of a cure. In contrast, low-income patients, particularly those with no income or relying on casual work or disability grants, face greater challenges, resulting in higher rates of adverse outcomes like LTFU or death during treatment [47]. Patients from low socioeconomic backgrounds are less likely to seek medical attention and have poor treatment outcomes [48]. Low income, unemployment, and a lack of social support all contribute to greater rates of LTFU and increased mortality while on treatment. According to [27], patients with low socioeconomic status are less likely to seek medical attention and have poor treatment outcomes. A study examining public healthcare expenditure from 1996 to 2016 found that despite increases in government health expenditure, the relationship between healthcare spending and economic development is complex. The research indicated that higher unemployment rates correlate with poorer healthcare performance, suggesting that economic challenges hinder effective healthcare delivery, particularly for diseases like TB [49]. Higher death rates among these patients emphasize the critical role of economic factors in treatment adherence and overall health. Additionally, social stigma and the lack of support systems in impoverished communities further complicate TB management. These findings suggest that effective TB control must address socioeconomic conditions through interventions like economic support, stigma reduction, and community-based support systems, alongside medical care, to improve treatment outcomes for vulnerable populations in rural Eastern Cape.

HCF 1 has a diverse distribution of DR-TB types, including notable proportions of Pre-XDR and XDR cases, alongside RR and MDR. HCF 3, 4, and 5 are dominated by RR-TB with few cases of other DR-TB types, indicating a less diverse distribution. HCF 2 and HCF 6 show a significant presence of both MDR-TB and RR-TB, reflecting a broader spectrum of drug resistance compared to HCF 4 and HCF 5. This suggests that RR-TB is the most prevalent type of DR-TB across all HCFs, which agrees with the study of Harling et al. [50], who also reported the prevalence of RR-TB. There are variations in the distribution of other types like MDR, Pre-XDR, and XDR, which highlights the ongoing challenge of managing drug resistance in TB [51]. These differences may reflect variations in patient populations, healthcare practices, or the stage of diagnosis at each facility. The population demographics and characteristics served by each HCF influence TB type distribution. Areas with higher poverty, overcrowding, or migration may have more cases of DR-TB due to poor living conditions and inconsistent healthcare access [52]. The facilities serving populations with higher HIV rates may see more severe TB forms, as HIV-positive individuals are more susceptible to developing DR-TB due to a weakened immune system [48]. Healthcare practices and resources significantly influence TB type distribution. Facilities with advanced diagnostic tools are better at identifying and classifying severe forms of DR-TB, leading to higher reported incidences of Pre-XDR and XDR-TB [53]. The facilities with strong treatment adherence support tend to have lower rates of DR-TB, as they can better ensure patients complete their treatment regimens, reducing the likelihood of resistance developing. Factors such as patient awareness, trust in healthcare providers, and support systems significantly influence treatment adherence, ultimately impacting the development of DR-TB [54,55]. Referral patterns and case complexity affect TB type distribution. Facilities managing severe cases often see higher rates of DR-TB, as they receive patients who have failed treatment elsewhere or have a history of inconsistent treatment. The history of TB treatment in an area influences the distribution of TB types. Areas with past treatment interruptions or inadequate TB control programs tend to have higher rates of DR-TB [56]. Additionally, inconsistent drug supply or the use of substandard medications can contribute to the development of drug resistance, impacting the prevalence of DR-TB in different facilities. The presence of more severe forms of drug resistance (Pre-XDR and XDR) in certain facilities like HCF 1 indicates potential hotspots that may require focused interventions and closer monitoring.

There are significantly lower survival rates among HIV-positive TB patients compared to those who are HIV-negative as seen in our study. The weakened immune system in people living with HIV is a major contributor to this disparity, highlighting the importance of early diagnosis and prompt initiation of treatment [34,57]. In a study conducted in KwaZulu Natal, the HIV-positive patients had a higher mortality rate (9.67%) compared to HIV-negative patients (2.91%) with the overall treatment success rate being lower for HIV-positive patients, indicating that HIV significantly affects TB treatment outcomes and survival rates in South Africa [28]. This emphasizes the need for strengthening integrated care strategies that address both HIV and TB simultaneously, including more aggressive treatment, closer monitoring, and additional patient support. TB treatment for HIV-positive individuals is more complex because they often need to manage both TB medications and antiretroviral therapy (ART) for HIV. Drug interactions between TB medications such as rifampicin and ART can reduce the effectiveness of both treatments, which necessitates careful selection and adjustment of drug regimens. A study that reviewed the management of individuals requiring ART and TB treatment in South African patients emphasized that rifampicin is a potent inducer of cytochrome P450 enzymes, which can reduce the plasma concentrations of non-nucleoside reverse transcriptase inhibitors (NNRTIs) and protease inhibitors (PIs). This reduction can lead to inadequate ART plasma concentrations and inferior treatment outcomes [58].

The underweight category had a high frequency of LTFU and treatment failure, while patients with normal BMI have better treatment outcomes. Underweight individuals experience social stigma, lack of support, or mental health issues that contribute to a higher likelihood of discontinuing treatment [59,60]. Nutritional deficiencies and a compromised immune system can lead to poorer responses to treatment and underweight patients may also have less physiological reserve to cope with illness, leading to complications [61]. A normal BMI is often associated with better overall health, which can facilitate more effective treatment responses and lower rates of complications. Research indicates that overweight patients generally experience a mild baseline disease severity and lower mortality rates compared to their underweight counterparts. This suggests that being overweight may confer some protective effects against severe outcomes related to TB, although the exact mechanisms remain under investigation [62,63,64].

This study highlights the significant challenges associated with managing TB in patients with comorbidities. Comorbidities complicate TB treatment by requiring multiple medications, which can interact negatively with TB drugs, leading to reduced treatment effectiveness, increased side effects, and higher mortality rates [65,66]. Patients often struggle with treatment adherence due to the complexity of managing multiple conditions, side effects, or the burden of taking many medications, increasing the risk of treatment failure. Comorbidities can also cause TB to progress more rapidly into severe forms, which are harder to treat and associated with higher mortality rates [65]. For example, HIV-positive individuals are more prone to developing extrapulmonary TB, which is harder to manage and has a higher risk of death. Mental health issues, such as depression and anxiety, further complicate TB treatment by reducing patients’ motivation to adhere to their medication regimen. Specific comorbidities like epilepsy and hypertension further complicate TB treatment. Epilepsy medications can interact with TB drugs, reducing their effectiveness and increasing the risk of seizures and drug resistance [66]. Hypertension, combined with TB, increases the risk of cardiovascular events and makes treatment adherence more difficult due to side effects and the need for frequent monitoring [66].

In evaluating the impact of previous drug history on treatment outcomes, patients with a history of prior treatment demonstrated higher rates of suboptimal outcomes, likely due to drug resistance—a recognized complication in those with prior drug exposure. Previous drug history and patient category have the strongest relationship, while treatment outcome is weakly correlated with these factors. Andriani and Yuliani’s meta-analysis supports the assertion that previously treated patients are at a higher risk for MDR-TB due to factors such as inadequate treatment supervision and inappropriate drug regimens [67]. The findings of Elduma et al. further substantiate this relationship, as they reported that 67.9% of MDR-TB patients had a history of previous TB treatment [68]. The treatment outcomes for MDR-TB patients are often less favorable, particularly when previous treatment failures are involved. Habibi et al. reported that the prevalence of MDR-TB is significantly higher following treatment failures, indicating that the history of treatment is a stronger predictor of poor outcomes [69].

## 5. Conclusions

This study highlights the complex interplay of socioeconomic factors, comorbidities, and healthcare access challenges in managing DR-TB in rural Eastern Cape. The findings underscore the critical need for integrated care strategies that address both medical and socioeconomic determinants to improve treatment outcomes. Higher mortality rates among patients with unstable income, comorbid conditions like HIV, and those facing treatment adherence challenges point to the necessity of focused interventions, including enhanced patient support, closer monitoring, and improved healthcare infrastructure. To effectively combat DR-TB in resource-limited settings, future efforts should focus on reducing socioeconomic disparities, ensuring comprehensive management of comorbidities, and strengthening the capacity of healthcare facilities. Addressing these issues holistically could lead to better patient outcomes and contribute significantly to the broader goal of controlling and eventually eradicating TB in high-burden regions.

Our study reports characteristics of DR-TB patients and the rates of drug resistance in the study population. Biological factors or aspects related to healthcare facilities or systems were not assessed, which limits our findings. Data collection relied solely on patient records, constrained by the information documented by clinicians, preventing us from evaluating other TB transmission factors like household air pollution. Additionally, incomplete data from retrospective patient records led to an imbalance in data collected and analyzed across healthcare facilities. Implementing quality control for data entry in patient files is essential. Only a limited number of cases were reviewed from HCF 5 due to challenges in timely access and data completeness. To address these limitations in future studies, several strategies could be implemented. First, expanding data collection sources beyond patient records to include interviews with patients or caregivers, household assessments, or environmental monitoring can capture critical information, such as household air pollution, which may not be routinely documented in clinical records. Additionally, a systematic process for real-time or regular data quality checks should be established, particularly for prospective studies.

## Data Availability

Data can be requested from the corresponding author.
